# Protective effect of unilateral vasospasm in the setting of HHH-associated posterior reversible encephalopathy syndrome: case report, review of the literature, and treatment considerations

**DOI:** 10.1186/s41016-018-0141-8

**Published:** 2018-12-03

**Authors:** Alankrita Raghavan, Jordan Xu, James M. Wright, Christina Huang Wright, Benjamin Miller, Yin Hu

**Affiliations:** 1 School of Medicine, Case Western Reserve University, 2109 Adelbert Road, Cleveland, OH 44106 USA; 2 Department of Neurological Surgery, University of California Irvine SOM, 1001 Health Sciences Rd, Irvine, CA 92617 USA; 3 Department of Neurological Surgery, University Hospitals Cleveland Medical Center, 11100 Euclid Avenue, Cleveland, OH 44106 USA; 4 Department of Neurology, University of Minnesota, 420 Delaware St SE, Minneapolis, MN 55455 USA

**Keywords:** Subarachnoid hemorrhage, Hyperdynamic therapy, Posterior reversible encephalopathy syndrome

## Abstract

**Background:**

Hyperdynamic therapy, also called triple-H therapy, is the standard treatment and prophylaxis for aneurysmal-associated vasospasm. In patients who are able to tolerate cardiopulmonary stressors induced by this therapy, it is of benefit as a modality for prevention and treatment of delayed ischemic neurologic deficit. However, it can be a cause of significant cardiopulmonary or neurologic sequelae. In rare cases, it can be associated with posterior reversible encephalopathy syndrome (PRES), secondary to prolonged vasopressor and hypertensive therapies.

**Case presentation:**

We present the case of a patient with right-sided aneurysmal-associated vasospasm who, after 10 days of triple-H therapy, experienced a seizure and was found to have left-sided PRES. Right-sided vasospasm served as a protective mechanism from triple-H therapy-associated PRES. It presented a treatment conundrum due to contradictory perfusion requirements. Hypertensive therapy was curtailed and in efforts to preserve local cerebral perfusion and vasodilation, local therapy with intrathecal nicardipine was initiated. We present our case, a review of the literature, and management considerations.

**Conclusions:**

Therapies that have conventionally functioned as second line treatments for aneurysmal subarachnoid hemorrhage (intra-arterial vasodilators and intrathecal vasodilators) may be beneficial as earlier treatments in the setting of vasospasm given the systemic difficulties and complications associated with HHH therapy in patients with PRES.

## Background

Hyperdynamic therapy is a conventional treatment for aneurysmal subarachnoid hemorrhage-associated cerebral vasospasm. Hypertension, hypervolemia, and hemodilution are used to ensure adequate cerebral perfusion in patients with severely constricted cerebral vasculature and abnormal autoregulation. Implementation of hyperdynamic therapy however does come with risks. Pulmonary edema, myocardial ischemia, hyponatremia, indwelling venous catheter-related complications, cerebral hemorrhage, and cerebral edema are all known sequelae [1]. A less commonly associated complication of triple-H therapy includes the development of posterior reversible encephalopathy syndrome (PRES). PRES can lead to disturbance of autoregulation, development of brain edema due to endothelial damage, and cerebral ischemia [2]. The development of PRES can be clinically confounding as presenting symptoms may be identical to delayed ischemic neurologic deficit (DIND). Management presents a significant conundrum, as treatments of these two pathologies are directly conflicting. We present a case of a patient who had complications both of clinical and angiographic right-sided vasospasm and triple-H induced PRES. On imaging, FLAIR changes were localized solely to her contralateral parieto-occipital hemisphere suggesting that her vasospasm was functioning as a protective mechanism against ipsilateral PRES. These two coinciding diagnoses created a treatment dilemma. In this article, we present our case, a review of the literature of triple-H-associated PRES and attempt to summarize the management of patients with both vasospasm and PRES from the literature and our experience.

## Case presentation

**Fig. 1:**
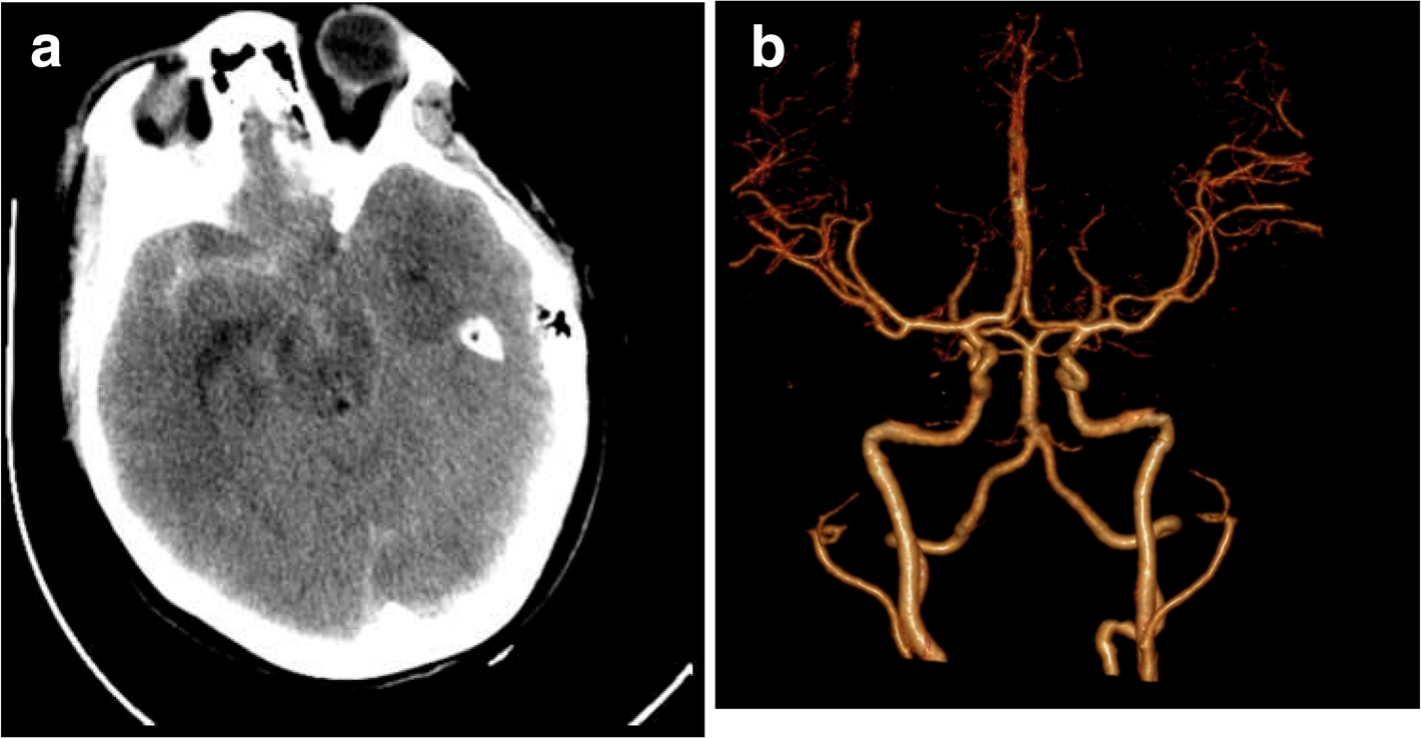
**a** Non-contrast CT head demonstrating the patient’s initial presenting scan with primarily right-sided subarachnoid hemorrhage. **b** CTA reconstructed images demonstrating normal bilateral vessel caliber

**Fig. 2:**
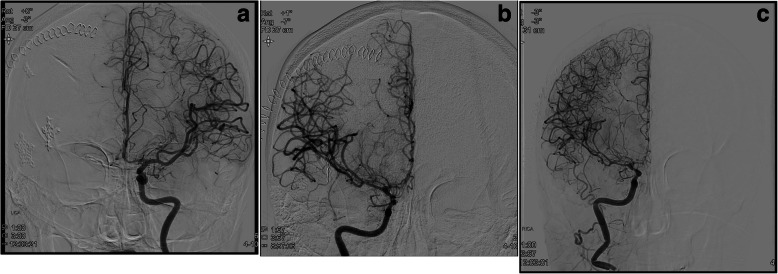
**a** Postoperative angiogram demonstrating clip occlusion. L ICA injection demonstrated no evidence of spasm. **b** R MCA and ACA concerning for mild non-flow-limiting spasm. **c** Repeat angiogram after neurologic deterioration demonstrates progressive narrowing of right MCA and ACA vasculature

A 33-year-old previously healthy woman presented after a sudden loss of consciousness and reported seizure. Upon arrival to the hospital, she complained of a moderate headache with severe neck pain with a Hunt Hess Score of 2. CT imaging demonstrated subarachnoid hemorrhage (Fig. 1a) from a right posterior communicating artery aneurysm. Importantly, no vasospasm was seen in the CTA from admission (Fig. 1b). She had an external ventricular drain (EVD) placed and underwent a craniotomy for clipping on post-bleed day 1. Post operatively, she was intact and monitored in the neurointensive care unit. Post-operative angiogram showed no evidence of flow-occluding spasm (Fig. 2a, b).

Her hospital course was complicated by severe vasospasm of the right anterior and middle cerebral artery. On post-bleed day 5, she began to experience visual auras and complain of blurry vision. Transcranial Doppler velocities were notably increased. She underwent endovascular intervention and administration of intra-arterial (IA) verapamil resulting in improved neurologic function. Triple-H therapy was initiated and her symptoms improved despite persistently elevated right transcranial doppler (TCD) velocities. On post bleed day 15, after 10 days of blood pressure augmentation to goal systolic blood pressures of 180 mmHg, she had an acute change in neurologic exam with a mixed aphasia and disorientation. Blood pressure was increased to a goal of 200 mmHg with no significant improvement. That same day, she experienced a complex partial seizure that progressed to a generalized tonic-clonic seizure. A repeat CTA demonstrated persistent right MCA spasm. An angiogram was performed (Fig. 2c), which showed vasospasm of the right ICA, MCA, and ACA, which was treated with IA verapamil. EEG did not demonstrate any subclinical seizure activity.

**Fig. 3:**
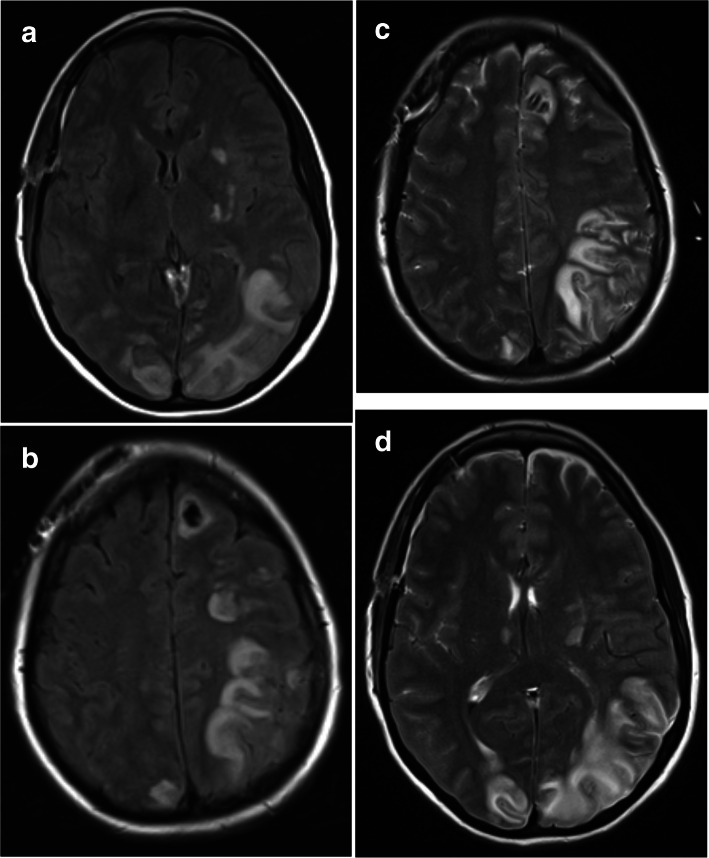
**a** Axial FLAIR demonstrating significant amount of predominantly left-sided parieto-occipital vasogenic edema. **b** Axial FLAIR demonstrating significant amount of predominantly left-sided parieto-occipital vasogenic edema. **c** T2 axial MRI demonstrating significant amount of predominantly left-sided parieto-occipital vasogenic edema. **d** T2 axial MRI demonstrating significant amount of predominantly left-sided parieto-occipital vasogenic edema

**Fig. 4:**
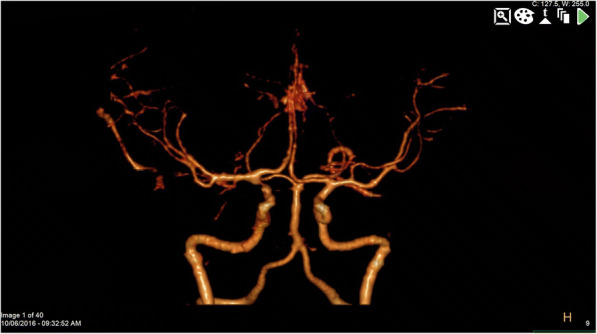
CTA reconstructions at 6 months post-hemorrhage demonstrating persistently occluded aneurysm and resolution of vasospasm

When her aphasia failed to resolve, an MRI of her brain was performed which showed left parieto-occipital vasogenic edema suggestive of PRES (Fig. 3). Hemodilution and hypervolemic therapy were discontinued. Her blood pressure parameters were relaxed to systolic blood pressure goals of 140–160 mmHg. Given the right-sided vasospasm and the left-sided PRES, the decision was made to administer intrathecal nicardipine through her EVD. She received 2 mg of nicardipine every 8 h for five doses. Her aphasia and confusion began to improve over the next few days. Vasopressors were discontinued and blood pressure goals relaxed. She was transferred out to the floor on day 26 and discharged to an acute rehab on day 28 with no focal neurological deficits. She followed up in the outpatient clinic after 2 months and was neurologically intact with intermittent headaches. She had no further seizures. A year after discharge, she remained neurologically intact and a CTA demonstrated complete resolution of her PRES-associated cerebral edema as well as no recurrence of her aneurysm or vasospasm (Fig. 4).

## Discussion

Hyperdynamic therapy is often utilized in prophylaxis and treatment of cerebral vasospasm after aneurysmal subarachnoid hemorrhage (aSAH) to prevent DIND. PRES is an uncommon but clinically significant syndrome that has been described in numerous case reports. The current hypothesis for a mechanism of PRES involves capillary leak in the setting of damaged endothelium. At high pressures, the intrinsic cerebral autoregulatory system fails and vessels are forcibly dilated leading to arteriolar distention and rupture of vascular endothelial cell connections. Extravasation of plasma proteins induces a vasogenic edema [3–5]. Etiologies include hypertension, pre-eclampsia, toxic drugs, immunosuppression, lupus, and renal failure. Symptoms vary widely, but most are often reversible with a reduction in blood pressure [6]. PRES typically localizes to the parieto-occipital region, an area believed to be less capable of autoregulation secondary to less sympathetic innervation [7]. Symptoms localize to the area affected and include altered mental status, seizures, visual changes, weakness, and if in the dominant hemisphere, aphasia.

Management of PRES and cerebral vasospasm together presents a treatment challenge. The treatment for each is in direct conflict with the pathophysiology of each other. We presented a patient with unilateral vasospasm of the right ACA and MCA, with complications of vasogenic edema arising in the contralateral hemisphere. While most reports of PRES demonstrate bilateral posterior occipito-parietal hemispheric involvement, a few case reports offer similar reports of unilateral vasospasm and contralateral vasogenic edema [2, 8]. As Dhar et al. proposed, this presentation is likely the result of persistent vasospasm on one side lowering perfusion pressure effectively presenting the development of PRES on the ipsilateral site. The unaffected hemisphere however, remains vulnerable to PRES-induced edema [8].

A literature review was performed of hyperdynamic therapy-associated PRES in patients with aSAH. The relative paucity of literature is likely secondary to the lack of standard MRI imaging performed in aneurysm patients. Awori et al. attempted to extrapolate common risk factors for patients with aSAH and triple-H-associated PRES. However, upon an extensive literature review, the cases are too few in number to establish significant risk factors [9]. As demonstrated in Table 1, PRES seems to occur more frequently in females and on average, occurs about 8 days after initiation of hyperdynamic therapy.

**Table 1:** Reports of PRES with aSAH-associated vasospasm

Author	Age, gender	Aneurysm location	Vasospasm location	Edema distribution	Treatment	Vasospasm treatment	Baseline BP (MAP)	Target BP (mmHg)	PRES (days after SAH)	PRES (days after HT)	Hx of HTN	mRS	Symptoms during PRES/CVS
Amin 1999 [10]	52, F	L MCA	Not described.	Bilateral occipital	Clipping	Hyperdynamic Dobutamine	150/70 (97)	SBP 200	13	7	N	1	Lethargy
	66, M	X		Bilateral occipital		Hyperdynamic Dobutamine	NA	SBP 200	12	10	Y	6	Lethargy aphasia Seizure R hemiparesis
Sanelli 2005 [11]	49, F	AComm	Bilateral ACA	Bilateral occipital R occipital ICH	Clipping	HHH	NA	SBP 140–200	17	13	NA	1	Lethargy
Wartenberg 2006 [12]	73, F	L ICA	L ACA and MCA	Bilateral occipital parietal	Clipping	HHH	145–175	SBP 140–200	10	7	Y	4	Coma with decorticate posturing
Jang 2010 [13]	65, F	PComm	Not described.	Bilateral temporal occipital	Coiling	HHH	130/80 (97)	SBP 165	7	1	N	1	Confusion, headache, and vision loss
Giraldo 2011 [14]	62, F	AComm	Circle of Willis and Bilateral ACA	Bilateral parietal occipital	Coiling	Hypertension	138/70 (93)	MAP 115	12	6	N	1	Confusion, headache, retro-orbital pain
	70, F	AComm	NA	Bilateral temporal occipital	Coiling	Hypertension Hypervolemia	149/58 (88)	MAP 120	12	11	Y	1	Lethargy seizure
	62, M	AComm	NA	Bilateral temporal occipital cerebellar	Clipping	Hypertension Hypervolemia	SBP 200	SBP 180	14	13	Y	4	Lethargy
Dhar 2011 [8]	47, F	R PComm	R MCA, bilateral ACA	L posterior temporal parietal	Clipping	Hypertension	MAP 65–75	MAP 120	13	7	N	1	Lethargy, confusion, L facial droop, aphasia, and HP
Voetsch 2011 [2]	35, F	R MCA	R MCA	Bilateral PCA L MCA	Clipping	Hypertension		MAP 110	7	4	NA	3	Headadche, seizure, and declined arousal
Awori 2016 [9]	63, M	AComm	Basilar, MCA, PCA, ACA	Bilateral temporal occipital	Clipping	Hypertension	140/109	MAP 110					Mental status change, seizure
Current Study	33, F	R PComm	R ACA and MCA	L parietal occipital	Clipping	HHH		SBP 180–200		10		0	Wernicke’s aphasia, seizure
Total	10, F; 3, M Avg age 57									Avg: 8 days		Avg mRS: 2.2	

*MAP* mean arterial pressure; *Hx OF HTN* history of hypertension (Y= Yes, N = No, NA = Not reported); *mRS* modified rankin score

Dhar et al. present a very nearly identical case of a patient with right sided vasospasm receiving triple-H therapy [8]. Workup for new confusion and aphasia included negative EEG and angiograms. MRI demonstrated significant left parieto-occipital FLAIR changes. Mean arterial pressure (MAP) goals were decreased from 110 mmHG to 70 mmHg with rapid resolution of her aphasia, hemiparesis, and orientation. She was discharged without neurologic deficits. Dhar et al. argue that, in such situations should concerns for DIND exist, more aggressive but focal therapies such as balloon angioplasty or intra-arterial vasodilators may be safer in this particular subset of patients [8]. The authors are in agreement with this theory and suggest that should clinical presentation of both PRES and DIND be a concern, angiographic intervention may be safer for the patient.

Voetsch et al. present a similar case of a patient with right MCA spasm and DIND requiring hyperdynamic therapy and intra-arterial nicardipine [2]. Augmentation of blood pressure led to increasing headaches, lethargy, and ultimately two generalized seizures. MRI demonstrated bilateral occipital and left parietal FLAIR changes. Hyperdynamic therapy was discontinued. The right MCA territory ischemia did increase in size over the course of her hospitalization, and the patient was discharged with a moderate left hemiparesis.

A review of the literature was performed to synthesize a possible treatment paradigm [2, 8, 10–14]. All authors initiated a relaxation of pressure parameters in their hyperdynamic therapy. Some reported immediate discontinuation of vasopressors while others performed a down-titration of vasopressors over several days. Most authors reported no occurrence of DIND upon a patient’s discharge. Voetsch et al. did report an increase in the size of one patient’s stroke territory after HHH therapy was discontinued [2].

Dhar et al. address the important concept that perhaps more focal treatments in the setting of PRES and significant vasospasm may be more beneficial than the conventional systemic treatments of HHH therapy [8]. Our patient underwent focal intra-arterial verapamil treatments and also received 48 h of intrathecal nicardipine in attempts to address both her vasospasm and her PRES. Though research remains to be performed, perhaps therapies that have conventionally functioned as second line treatments (intra-arterial vasodilators and intrathecal vasodilators) may be beneficial as earlier treatments in the setting of vasospasm given the systemic difficulties and complications associated with HHH therapy.

A review of the English literature found eight studies that reported on the use of intraventricular or cisternal administration of nicardipine for the treatment of aneurysmal SAH-associated vasospasm (Table 2). Few of these studies were sufficient in number and outcomes data to know when in the treatment algorithm, intra-thecal nicardipine should be implemented. These studies show however that the use of nicardipine is not associated with significant complications. Rates of ventricular-associated infections or meningitis varied and were not consistently reported. There is some evidence that TCD velocities are improved and maintain this improvement after administration [15, 16]. In patients where traditional HHH therapy may be contraindicated including patients with PRES, this treatment modality can function as a useful resource. Anecdotally, some patients experience nausea and emesis associated with administration. Others are also unable to tolerate prolonged EVD clamp times after administration of the drug.

**Table 2:** Summary of studies that have utilized intraventricular or cisternal nicardipine for the treatment of aSAH-associated vasospasm

Author	Study type	Vasopasm diagnosis	IVTN treatment	Patients (control)	Aneurysm	HH grade	Fisher grade	Aneurysm treatment	Results	Patient outcomes	Complications
Acute treatment for vasopasm											
Ko 2016 [16]	Retrospective case series Study of hemodynamic changes during intraventricular nicardipine treatment (IVTN) in refractory vasospasm	Multimodality monitoring, brain oxygen tension, CBF, brain metabolism	4 mg, q8 nicardipine solution Clamp EVD for 1 h Mean 4.9 doses/patient	11 (0) Age mean: 49 8 female 3 male	NA	IV: 5 V: 6	3: 7 pt. 4: 4 pt	NA	*Mean ICP increased slightly (2.5 ± 0.9 mmHg), peaking at 20 m *ICP decreased 20–30 m after injection (3.7 ± 1.8 mmHg) *MAP, PBO2, CBF, autoregulation indices did not change significantly	3 month follow up mRS 5: 8 patients mRS 6: 3 patients	Pneumonia (2) Seizures (2) Sepsis (2) Globala cerebral edema (11) Hydrocephalus (8) MI (2)
Lu 2012 [15]	Retrospective case-control Monitored IVTN effects with TCD	TCDs	4 mg Median 7 doses/patient (range 1–17)	14 (14)** Age mean: 45 12 female 2 male	ACOM: 3 PICA: 2 PCOM: 2 MCA: 2 Pericallosal: 1 Vertebral: 1 VB: 1 ICA: 1 ACA: 1	NA	3: 3 pt. 4: 11 pt	Coil: 8 Clip: 5 Stent: 1	*Mean flow velocity decreased after IVTN (R MCA: 120.2—> 82.0 cm/s, L MCA: 101.6—> 72.8 cm/s) *No significant difference in clinical outcomes	No significant difference between control and treatment group at 30 and 90 days	No bleeding or infection incidents.
Webb 2010 [24]	Retrospective case series TCD measurement of changes post-IVTN	TCDs	4 mg q8–12 Clamp EVD for 30 m Mean 6.7 doses/patient	64 (0) Age mean: 52	NA	I, II: 13 III: 30 IV, V: 21	2: 6 pt. 3: 45 pt. 4: 13 pt	Coil: 35 Clip: 29	*IVTN reduced mean flow velocity by 26.3 cm/s in MCA and 7.4 cm/s in ACA, maintained over 24 h with continued administration *No change in ICP	Not described.	Ventricular-related infection: 4 clinically proven, 7 clinically possible
Ehtisham 2009 [23]	Retrospective case series Vasospasm refractory to standard medical and endovascular treatment.	TCDs	4 mg q12; stopped once MCA velocity < 120 cm/s Clamp EVD for 30 m	6 (0) Age mean: 45 5 female 1 male	PCA: 2 ACA: 1 PICA: 1 VB: 1 Pericallosal: 1	II: 1 III: 2 IV: 2 V: 1	3: 2 pt. 4: 4 pt	Coil: 3 Clip: 3	*IVTN reduced MCA flow velocity by 43.1 ± 31.0 cm/s	Not described.	No major infection or reverse reaction.
Goodson 2008 [25]	Retrospective case series IVTN used in refractory vasospasm	Symptomatic	4 mg q12 Clamp EVD for 1 h Length of treatment 9.5 days (5–17)	8 (0) Age mean: 51 7 female 1 male	ACA: 3 ACOM: 3 MCA: 1 PICA: 1	I: 4 II: 2 III: 2	4: 8 pt	Coil: 5 Clip: 3	*IVTN well-tolerated with minimal side effects	*7 moderate to good outcomes *1 patient died in ICU *Median modified rankin score: 2 (2–6)	1 had nausea and headache
PROPHYLACTIC TREAMENT FOR VASOSPASM											
Barth 2009 [26]	Prospective trial Intraventricular nicardipine prolonged release implants (NPRI)	Angiogram or CT angiogram	6 or 10 pellets, 4 mg/pellet	31 (16)** Age mean: 52 20 female 11 male	ACA: 15 PCOM: 5 MCA: 4 VB: 2 PICA: 2 Pericallosal: 2 ICA: 1	I: 6 II: 11 III: 7 IV: 7	NA	Clip: 17 Coil: 14	*NRPI had larger mean diameter on DSA (90 ± 24% vs 80 ± 30% control) *NPRI group had less moderate/severe vasospasm (41% vs 73% control) *Effect not seen in coil group *No difference in dose	Not described.	No different from control group
Suzuki 2001 [22]	Prospective trial Post operative intrathecal nicardipine	Symptomatic angiogram	4 mg, q12 on post-op days 3–14 (via cisternal drain)	177 (0) Age mean: 59 121 female 56 male	ICA: 66 ACOM: 58 MCA: 43 ACA: 6 VB: 4	I: 11 II: 112 III: 35 IV: 16 V: 2	NA	Clipped: 177	*20 (11.3%) with angiographic vasospasm *10 (5.7%) with symptomatic vasospasm *Low rates compared to literature (aVS 19–97%, sVS 5–90%)	mRS 2–3: 89.2% at 6 months	11 (6.2%) meningitis
Shibuya 1994 [21]	Prospective trial Post operative intrathecal nicardipine	Symptomatic angiogram	2 mg, q8 for 10–14 days (via cisternal drain)	50 (91)** Age mean: 54	ACA: 23 ICA: 13 MCA: 14 VB: 0	I: 0 II: 15 III: 25 IV: 10	NA	Clipped: 50	*Symptomatic vasospasm decreased by 26% *Angiographic vasospasm decreased by 20% *Neither are statistically significant *Increased ‘good clinical outcome’ at 1 month post-bleed by 15%	Not described.	2 (4%) meningitis 4 (8%) hydrocephalus requiring shunts

^**^Historical controls; *VS* vasospasm; *IVTN* intraventricular nicardipine, *PBO2* brain oxygen tension, *CBF* cerebral blood flow, *HH* Hunt Hess, *TCDS* transcranial dopplers study, *M* male, *F* female

Currently other agents including intravenous endothelin-1 receptor antagonists and intrathecal nimodipine, and milrinone are being studied as agents for the treatment of vasospasm. Endothelin receptor antagonists demonstrated potential in animal studies with reduction rates of vasospasm and increase in average vessel diameter; however, an updated meta-analysis has found that it does not demonstrate overall improved outcomes [17]. Levosimendan is a calcium channel sensitizer that has recently been shown to antagonize prostaglandin-induced vasoconstriction and upregulate the nitric oxide-cyclic guanosine monophosphate pathway thereby inducing increased vasorelaxation. Animal models have yet to be studied [18]. Intrathecal milrinone demonstrated a successful safety and feasibility study in a study of 170 patient receiving lumbar subarachnoid milrinone injections, but further trials are required to validate this protocol [19, 20]. Intrathecal nimodipine, nicardipine, and milrinone are not yet standards of care and have not yet been proven to improve overall outcomes in conventional research. However, these therapies have demonstrated utility in specific cases of patients where the use of conventional therapeutics, including HHH therapies, lead to unwelcome complications. Intrathecal administration of nicardipine demonstrates very little systemic effects [21–23].

## Conclusion

This case report and literature review suggest that the management paradigm of aSAH in the context of PRES should shift towards more focal modalities including intra-arterial vasodilators and intrathecal agents before reliance on HHH therapies due to the possibility of cardiopulmonary and neurologic complications. Early diagnosis of PRES in neurosurgical patients is essential for therapy modification to ensure better outcomes.

## Abbreviations

ACA: Anterior cerebral artery
DIND: Delayed ischemic neurologic deficit
EVD: External ventricular drain
HHH: Hypertension, hypervolemia, and hemodilution
IA: Intra-arterial
ICA: Internal carotid artery
MAP: Mean arterial pressure
MCA: Middle cerebral artery
PRES: Posterior reversible encephalopathy syndrome
aSAH: Aneurysmal subarachnoid hemorrhage
TCD: Trans-cranial doppler
